# Myelotoxicity and kidney dysfunction related to the use of trimethoprim-sulfamethoxazole for the treatment of *Pneumocystis jirovecii* pneumonia: a case report of severe adverse events with a common drug

**DOI:** 10.1590/S1678-9946202466018

**Published:** 2024-03-18

**Authors:** Isabel Cristina Melo Mendes, Roxana Flores Mamani, David Richer Araujo Coelho, Clarisse Pimentel

**Affiliations:** 1 Instituto Estadual de Infectologia São Sebastião, Rio de Janeiro, Rio de Janeiro, Brazil; 2 Universidade Federal do Rio de Janeiro, Programa de Pós-Graduação em Doenças Infecciosas e Parasitárias, Rio de Janeiro, Rio de Janeiro, Brazil; 3 Instituto Nacional de Infectologia Evandro Chagas, Manguinhos, Rio de Janeiro, Brazil

**Keywords:** Adverse events, Myelotoxicity, Thrombocytopenia, Cotrimoxazole, Trimethoprim, Sulfamethoxazole

## Abstract

Trimethoprim-sulfamethoxazole (TMP-SMX) is the primary therapeutic option for *Pneumocystis jirovecii* pneumonia (PCP). Gastrointestinal symptoms and cutaneous rash are common side effects, with hyperkalemia being uncommon in patients without kidney dysfunction, and myelotoxicity being even rarer. We present the case of a male patient with hypertension and a recent diagnosis of non-Hodgkin lymphoma, undergoing rituximab treatment for two months. He was admitted to the intensive care unit due to dyspnea, tachypnea, and pleuritic pain, requiring mechanical ventilation. Chest computed tomography showed bilateral and multilobed ground-glass opacities, compromising more than 80% of the lung parenchyma. Pulmonary tuberculosis and COVID-19 were ruled out. An angiotomography and Doppler ultrasound revealed an extensive pulmonary thrombus and deep venous thrombosis. Empiric treatment with TMP-SMX for PCP was initiated, but within four days, the patient experienced metabolic acidosis and severe hyperkalemia, necessitating hemodialysis. He also presented with progressive pancytopenia and critical levels of leukopenia and thrombocytopenia. The hypothesis of TMP-SMX-induced myelotoxicity was suspected. Considering the unavailability of an alternative treatment, it was opted to continue TMP-SMX and initiate a granulocyte-colony-stimulating factor. However, the patient maintained medullary deterioration, becoming refractory to the transfusion of blood derivates. On the 17^th^ day of treatment, a clinical decision was made to suspend TMP-SMX, leading to improvements within 48 hours in marrow and kidney functions, metabolic acidosis, and hyperkalemia. Despite all efforts, the patient died after 35 days of hospitalization due to hospital-acquired infections. This case highlights the importance of clinicians recognizing potential myelotoxicity with TMP-SMX and promptly discontinuing the drug if necessary.

## INTRODUCTION

Trimethoprim-sulfamethoxazole (TMP-SMX) is an antimicrobial combination used in various clinical settings since its release in the 1960s. It acts by inhibiting bacterial synthesis of tetrahydrofolic acid, the active form of folic acid and a necessary cofactor for the synthesis of nucleobases for bacterial DNA. The combined use of its two components results in a sequential blockade, with *in vitro* synergism and an effective bactericidal action^1^.

It has a broad spectrum of activity, targeting enterobacteria, *Staphylococcus* spp., and *Streptococcus* spp. With the rise of antimicrobial resistance in the last decades, its role in treating bacterial infections empirically has diminished. However, it remains an important option for less prevalent pathogens, such as *Stenotrophomonas maltophilia, Nocardia* spp., *Isospora* spp., *Burkholderia pseudomallei*, and *Pneumocystis jirovecii* ^1,2^.

Although TMP-SMX has a favorable safety profile and is well-tolerated for most people, some individuals may experience several adverse events, including gastrointestinal, cutaneous, renal, hematological, and psychiatric symptoms^1^. In this article, we present a case where an immunosuppressed patient experienced severe hyperkalemia due to this drug, which was resistant to clinical treatment and led to the need for hemodialysis and subsequent myelotoxicity.

This case report was approved by the local Research and Ethics Committee (CAAE Nº 48749021.3.0000.5252, Plataforma Brasil).

## CASE REPORT

A 47-year-old male patient with a medical history of untreated hypertension and non-Hodgkin lymphoma was admitted to the intensive care unit due to dyspnea, tachypnea, and pleuritic pain. He had undergone chemotherapy two years prior and had started maintenance therapy with rituximab two months before admission. His respiratory distress intensified, requiring mechanical ventilation four days post-admission. Initial laboratory tests indicated moderate anemia (Hb 8.1 mg/dL, Ht 24.9%) and leukopenia (white blood cell count 2,800 cells/dL), with normal platelet count (215,000 cells/dL), and mild renal dysfunction. Notably, serum potassium and sodium levels were within the normal range (U 62 mg/dL, Cr 1.3 mg/dL, K 4.7 mEq/L, Na 136 mEq/L). An arterial blood gases analysis revealed normal serum bicarbonate and lactate levels associated with hypocapnia (HCO_3_ 21 mmol/L; lac 0.7 mmol/L; pCO_2_ 29.4 mmHg). Chest computed tomography showed bilateral and multilobed ground-glass opacities, compromising over 80% of the lung parenchyma. Both pulmonary tuberculosis and COVID-19 were ruled out after negative results of acid-fast bacilli and MTB GeneXpert of tracheal secretion and negative antigen test in nasopharyngeal swab, respectively. An angiotomography and Doppler ultrasound revealed an extensive right pulmonary thrombus and right deep venous thrombosis.

Empiric treatment with TMP-SMX for *Pneumocystis jirovecii* pneumonia (PCP) was initiated. Specific diagnostic exams, such as molecular tests or culture, were not available in the unit, but considering the clinical and radiological features, the context of immunosuppression, and the exclusion of other pulmonary infections, PCP was not ruled out. The patient also presented an elevated serum LDH (772 U/L), strengthening the hypothesis of PCP.

However, within four days, the patient experienced metabolic acidosis and severe hyperkalemia. Despite being considered the probable cause, TMP-SMX was maintained due to the unavailability of primaquine, one of the components of the alternative treatment registered in Brazil. These alterations were, therefore, treated with conservative measures—isotonic solution of sodium bicarbonate, diuretics, and gastrointestinal chelating agents. In addition, potassium serum levels were closely monitored, but the patient’s kidney function continued to deteriorate, with his creatinine levels going from 1.6 mg/dL to 2.5 mg/dL and requiring the initiation of hemodialysis on the 14^th^ day post-admission (stage 3 AKI according to KDIGO classification^3^).

Concurrently, the patient exhibited progressive pancytopenia. The hypothesis of TMP-SMX-induced myelotoxicity was suspected, but, due to the same reason considered for hyperkalemia, the drug was not suspended, and a granulocyte-colony-stimulating factor (G-CSF) was started. Nevertheless, the patient’s marrow function continued to decline, becoming refractory to the transfusion of blood derivates and presenting with critical levels of leukopenia and thrombocytopenia. On the 17^th^ day of treatment, TMP-SMX was suspended, leading to an improvement in marrow and kidney functions, metabolic acidosis, and hyperkalemia over the following 72 h (Table 1). The patient’s condition was further complicated by multiple hospital-acquired infections from resistant pathogens, including *Acinetobacter baumannii, Proteus* sp., *Pseudomonas aeruginosa*, and methicillin-resistant *Staphylococcus aureus* (MRSA). Despite comprehensive medical interventions, the patient died after 35 days of hospitalization.

**Table 1:** Laboratory values describing blood cell counts, kidney function and electrolytes throughout hospitalization.

DH	D1	D6	D7	D8	D15	D17	D18	D19	D20	D22
Hb	8.1	6.1	6.8	6.8	6.0	6.7	7.7	7.2	8.0	6.1
Ht	24.9	21.9	20.8	20.5	18.1	20.1	23.0	21.8	23.1	19.0
WBC	2,800	2,600	1,900	1,600	800	900	3,500	3,500	6,600	33,000
Plat	215,000	257,000	242,000	259,000	77,000	33,000	40,000	34,000	52,000	91,000
U	62	66	-	57	-	98	108	178	98	142
Cr	1.6	1.5	-	1.2	-	2.5	2.6	3.5	2.2	3.1
Na	136	135	134	135	-	133	130	134	138	139
K	4.7	4.3	6.9	6.3	-	4.8	3.6	4.4	4.7	4.8

DH = day of hospitalization; Hb = hemoglobin (g/dL); Ht = hematocrit (%); WBC = white blood cell count (cells/mm^3^); plat = platelets (cells/mm^3^); U = urea (mg/dL); Cr = creatinine (mg/dL); Na = sodium (mEq/L); K = potassium (mEq/L).

## DISCUSSION

In this case report, we describe a non-HIV immunosuppressed male patient who developed severe hyperkalemia refractory to medical treatment. This condition led to the need for renal replacement therapy (hemodialysis). Additionally, the patient exhibited myelotoxicity that was a result of cotrimoxazole use during a severe PCP presentation that required mechanical ventilation. TMP-SMX is a widely prescribed antibiotic worldwide. Resulting from the combination of two antibiotics—trimethoprim (a diaminopyrimidine) and sulfamethoxazole (a sulfonamide)—its broad spectrum of activity favors its use in several clinical settings. Notably, TMP-SMX is the primary treatment for *Pneumocystis jirovecii* pneumonia, a potentially severe opportunistic infection predominantly affecting immunocompromised individuals. TMP-SMX is considered a safe drug with a well-known profile of adverse events. In immunocompetent patients, gastrointestinal, more commonly nausea, vomiting, anorexia, and dermatological reactions, such as maculopapular rash, urticaria, diffuse erythema, and morbilliform lesions, are the most frequent and tend to be mild and reversible, usually not requiring treatment discontinuation. In immunosuppressed patients, most studies were conducted with people living with HIV and have shown a higher incidence of adverse events when compared with the immunocompetent population. Up to 50% of immunocompromised patients might require drug discontinuation due to toxicities^1^.

While hyperkalemia is an expected, well-described adverse event, its occurrence is considered uncommon. Early studies have suggested that it was more frequent with high doses of TMP-SMX^4,5^, but other reports have demonstrated that it can also occur with the lower doses often used to treat common bacterial conditions, such as urinary tract or skin and soft tissue infections, or even for prophylaxis, especially in the presence of concomitant renal insufficiency^1,6^. Myelotoxicity is a rare but potentially life-threatening event. Sulfonamides have been associated with hematological disorders, including several forms of anemia, granulocytopenia, agranulocytosis, and thrombocytopenia. The rates of these adverse events reported with TMP-SMX are considered similar to the ones reported with other sulfonamides, supporting the hypothesis that sulfamethoxazole is the culprit component. Different from other side effects, myelotoxicity does not seem to be dose-related^1,7^.

Our patient presented with both hyperkalemia and bicytopenia. The prescribed elevated doses for PCP treatment, as per national guidelines^8^, coupled with his kidney dysfunction, made him particularly prone to hyperkalemia^1^. An interesting feature of our case was the severity and resistance to potassium correction measures, leading to the need for renal replacement theraphy.

Typically, addressing non-chemotherapy drug-induced cytopenia involves withdrawal of the suspected drug and implementing preventive measures against infections. The use of G-CSF has been shown to reduce the duration of neutropenia in patients with agranulocytosis^7^. Transfusion of blood components might also be necessary. For our patient, the severity of the respiratory infection and the unavailability of a viable alternative treatment with primaquine and clindamycin precluded the discontinuation of TMP-SMX. Even with several days of CSF, his neutrophil counts did not improve. He also developed severe thrombocytopenia, which contraindicated the administration of anticoagulants—a necessary approach for his confirmed pulmonary embolism—due to the high risk of bleeding. Only after suspension of TMP-SMX that all these side effects diminished.

One could hypothesize that our patient’s hematological abnormalities would be due to rituximab-induced late-onset neutropenia (R-LON), a rare adverse event related to the use of this anti-CD20 biological agent.

The R-LON is defined as an absolute neutrophil count of less than 1.5 × 10^9^cells/mm^3^, occurring four or more weeks after the last administered dose of rituximab in the absence of previous neutropenia and without other possible related causes. Time before onset has been reported to range from 38 to 175 days^9^, with an average duration of 5 to 77 days^10^. The exact incidence of R-LON is unknown, and so is its pathophysiology^11^, but its prevalence is estimated at 1.3 to 27%, depending on the underlying disease. In most cases, it has a benign course with a spontaneous resolution^9^. There is a risk for the development of infections, but these are usually mild^10^.

Although R-LON can manifest several months post-rituximab administration, our patient’s rapid hematological and renal recovery after discontinuation of TMP-SMX strongly suggests TMP-SMX as the primary causative agent for both alterations observed during his hospitalization.

## CONCLUSION

TMP-SMX is a very frequently used antimicrobial and the primary treatment for potentially fatal diseases, such as *Pneumocystis jirovecii* pneumonia. Although usually safe, some adverse events can be life-threatening, and adequate monitoring must be undertaken, especially in immunosuppressed patients who have a higher risk of developing drug-related toxicity. Clinicians should be aware of the possibility of myelotoxicity and hyperkalemia with the use of TMP-SMX to promptly discontinue the drug if necessary.
